# Prognostic Model to Predict Cancer-Specific Survival for Patients With Gallbladder Carcinoma After Surgery: A Population-Based Analysis

**DOI:** 10.3389/fonc.2019.01329

**Published:** 2019-12-12

**Authors:** Chaobin He, Zhiyuan Cai, Yu Zhang, Xiaojun Lin

**Affiliations:** ^1^State Key Laboratory of Oncology in South China, Department of Hepatobiliary and Pancreatic Surgery, Collaborative Innovation Center for Cancer Medicine, Sun Yat-sen University Cancer Center, Guangzhou, China; ^2^State Key Laboratory of Ophthalmology, Zhongshan Ophthalmic Center, Sun Yat-sen University, Guangzhou, Guangdong, China

**Keywords:** gallbladder carcinoma, cancer-specific survival, overall survival, nomogram, prognosis

## Abstract

Predicting the prognosis of gallbladder carcinoma (GBC) has always been important for improving survival. The objective of this study was to determine the risk factors of survival for patients with GBC after surgery and to develop predictive nomograms for overall survival (OS) and cancer-specific survival (CSS) using a large population-based cohort. We identified 2,762 patients with primary resectable GBC in the Surveillance, Epidemiology, and End Results (SEER) database for the period of 2004 to 2014 and another 152 patients with GBC after surgery from Sun Yat-sen University Cancer Center (SYSUCC) for the period of 1997 to 2017. The 1-, 2-, and 3-year cancer-specific mortalities were 37.2, 52.9, and 59.9%, while the competing mortalities were 5.8, 7.8, and 9.0%, respectively. Nomograms were developed to estimate OS and CSS, and these were validated by concordance indexes (C-indexes) and evaluated using receiver operating characteristic (ROC) curves. The C-indexes of the nomograms for OS and CSS prediction were 0.704 and 0.732, respectively. In addition, compared with the 8th Tumor-Node-Metastasis staging system, the newly established nomograms displayed higher areas under the ROC curves for OS and PFS prediction. The nomograms are well-validated and could thus aid individual clinical practice.

## Introduction

Biliary tract cancers are relatively rare tumors globally; however, these diseases often carry a poor prognosis (1, 2). Gallbladder carcinoma (GBC), which is the most common biliary tract cancer, is traditionally regarded as a highly lethal disease with a 5-year survival rate of <5% (3). The majority of patients are not suitable candidates for curative resection (4), due to the nonspecific symptoms and highly invasive character of this disease (5). Most of the clinical trials focused on advanced diseases of GBC while no obvious breakthroughs were achieved (6, 7). It was widely accepted that surgery provided the best option for patients to improve survival. However, even after complete resection, locoregional recurrence is common, and the prognosis is unsatisfactory. Adjuvant therapies after surgery were also inevitable (8, 9). Therefore, to further select patients with risk factors after surgery and who were more suitable for adjuvant therapies, there is thus considerable interest in exploring the potential benefits of a staging system that can be used to predict the prognoses and subsequent treatment after surgery for patients with GBC. However, most previous studies have provided limited information due to the limited number of patients (10, 11). Most staging systems, including the 8th edition of the Tumor-Node-Metastasis (TNM) staging system of the American Joint Commission on Cancer (AJCC) (12), depend entirely on the final stratified pathological stage and only consider the anatomic characteristics of the tumor, ignoring the impact of other prognostic factors, such as age, gender, and tumor grade. Moreover, most studies have focused on the separate outcomes of the prognostic factors for patients with GBC (10, 13) and have thus failed to show the effects of the mutual influences among the prognostic factors. It is therefore necessary to establish a more effective and accurate staging system to predict the prognoses of patients with GBC.

Many patients who are older than 60 years face high rates of comorbidities (14, 15). Compared with healthy populations, increased morbidity and mortality, which are related to these comorbidities, coexisting pulmonary disease, heart disease, and liver disease, are observed in patients with GBC (16). Deaths caused by these comorbidities, other than original tumors, increases as age increases (17). It is therefore important to consider such risks when evaluating their prognoses. However, in most previous studies, only overall survival (OS) on the basis of the Kaplan–Meier and Cox methods was evaluated. Accordingly, misleading conclusions were easily reached since the presence of competing risks was not recognized and evaluated in the survival analyses (18). Due to the unique feature to evaluate the informative nature of the censoring, consideration of competing risk methods can allow for more accurate evaluations of prognoses and should be included in survival analyses (19, 20). Based on the data contained in the Surveillance, Epidemiology, and End Results (SEER) database, the present study aimed to evaluate the competing risks and to develop nomograms to investigate OS and cancer-specific survival (CSS) in patients with GBC after surgery.

## Materials and Methods

### Patients

Patients who had been diagnosed with GBC and had received resections from 2004 to 2014 were identified retrospectively in the SEER database. In addition, consecutive patients with pathological diagnosis of GBC after surgery between 1997 and 2017 at the Department of Hepatobiliary and Pancreatic Surgery of Sun Yat-sen University Cancer Center (SYSUCC) were also enrolled in this study. All included patients had received radical surgical resection and were pathologically confirmed adenocarcinoma. The following classifications were confirmed: the International Classification of Diseases for Oncology, Third Edition (ICD-O-3), the histology code 8140 and the ICD-O-3 site code C23.9. The exclusion criteria have been described in a previous study (21), which mainly included (1) primary multiple tumors, (2) missing or incomplete information of follow-up, (3) pathologically confirmed not adenocarcinoma, (4) in-hospital or 30-day mortality after surgery, (5) American Society of Anesthesiologists (ASA) Physical Status 3 or above levels (14). Patients from the SEER dataset were randomly selected to serve as the training and internal validation cohorts in a ratio of 3:1. Patients selected from the SYSUCC dataset served as an external validation cohort in this study.

### Data Collection

Clinical and pathological variables, including age, gender, tumor size and grade, TNM stage, follow-up information, and cause of death, were collected and analyzed. Lymph node ratio (LNR) was defined and calculated according to our previous study (21). OS and CSS were defined as the duration from the date of diagnosis to the last follow-up or death due to all causes or GBC, respectively.

### Statistical Analysis

Cancer-specific death and non-cancer-specific death were regarded as two competing events. Gray's test was used to compare the differences in the cumulative incidence function (CIF), which was used show the probability of each event between the groups (22). OS was analyzed using the Kaplan–Meier method, and the survival differences were compared using a log-rank test. Significant predictors of OS were determined by multivariate analyses and used to construct nomograms based on the Cox regression model. The Fine and Gray model was also utilized to determine the prognostic factors and to construct the competing risk nomograms (23). Concordance indexes (C-indexes) and calibration curves were used to measure the performance of the nomograms (24, 25) while ROC curves were used to compare the precision of the survival prediction of the nomograms and the 8th TNM staging system. All the statistical analyses were performed using R version 3.4.2 software (R Foundation for Statistical Computing, Vienna, Austria. http://www.r-project.org). Statistical significance was defined as a two-tailed *p* < 0.05.

## Results

### Patient Characteristics

A total of 2,762 patients with GBC were identified retrospectively from the SEER database and another 152 patients with GBC after surgery were also included from the SYSUCC dataset in this study. Overall, 71.8% of the patients were female, and white people made up 74.8% of the total study population. A large proportion of the patients (76.6%) were older than 60 years. More than half of the tumors were smaller than 3 cm, and moderate differentiation (*n* = 1,209, 43.8%) was the most common tumor grade. A total of 870 (31.5%) patients had lymph node (LN) metastasis, and 70 (2.5%) patients had four or more metastatic LNs.

**Figure 1:**
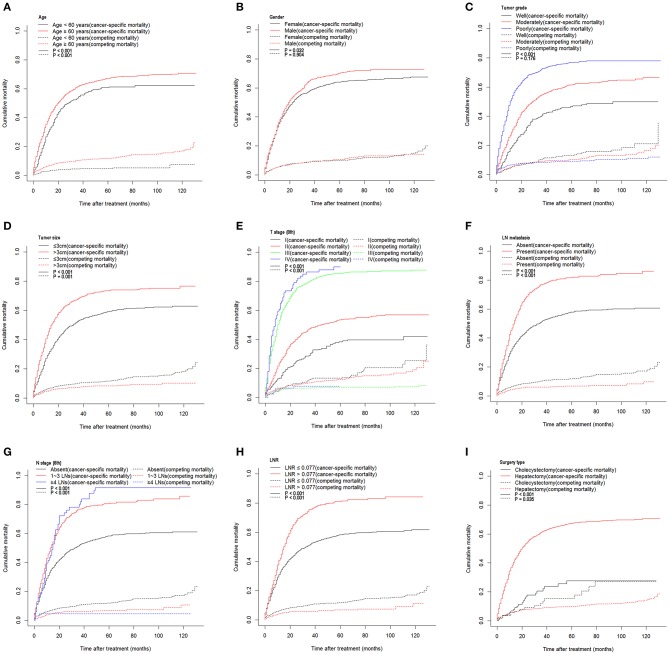
Cumulative cancer-specific and competing mortality stratified by patient characteristics: **(A)** age; **(B)** gender; **(C)** tumor grade; **(D)** tumor size; **(E)** T stage (8th); **(F)** LN metastasis; **(G)** N stage (8th); **(H)** LNR; **(I)** surgery type. LN, lymph node; LNR, lymph node ratio.

A total of 1,760 deaths were observed during the follow-up period of 11 months (range, 1–131 months), of which 1,502 were cancer-specific deaths and 258 deaths were from other causes. The corresponding CIF curves are shown in Figure 1. With a cutoff value of 0.077, the LNR was associated with the optimal Youden index for prognosis prediction. The cumulative probability of death from GBC increased with increasing age at diagnosis. The cumulative incidences of competing causes of death also increased with increasing age. Significantly higher probabilities of death were observed in the male than in the female patients. Patients with characteristics of smaller tumor size, earlier T stages (8th), the absence of LN metastasis, earlier N stages (8th), lower LNR values, and earlier TNM stages (8th) were all less likely to die as a result of GBC and more likely to die as a result of competing causes. Compared with well-differentiated tumors, moderately or poorly differentiated tumors contributed to the more aggressive effect of death from GBC, while tumors with different tumor grades did not exhibit considerable differences in terms of competing causes (Table 1).

**Table 1:** Overall survival rates and cumulative incidences of mortality among patients with gallbladder carcinoma.

**Characteristic**		**Patients**		**Overall survival rate (%)**			***P***	**Cancer-specific mortality (%)**			***P***	**Non-cancer-specific mortality (%)**			***P***
		**No**.	**%**	**1 year**	**2 years**	**3 years**		**1 year**	**2 years**	**3 years**		**1 year**	**2 years**	**3 years**	
Total		2762	100	57.0	39.3	31.1		37.2	52.9	59.9		5.8	7.8	9.0	
Age (years)	<60	647	23.4	66.9	48.4	41.2	<0.001	30.2	47.6	54.4	<0.001	2.9	4.1	4.3	<0.001
	≥ 60	2115	76.6	54.0	36.6	28.1		39.3	54.4	61.6		6.7	9.0	10.3	
Gender	Female	1984	71.8	57.4	40.3	33.0	<0.001	36.8	51.9	58.1	0.032	5.8	7.8	8.9	0.904
	Male	778	28.2	55.9	36.7	26.0		38.1	55.3	64.9		6.0	7.9	9.1	
Tumor grade	Well	406	14.2	75.7	60.3	49.3	<0.001	18.8	32.2	40.2	<0.001	5.5	7.5	10.5	0.176
	Moderate	1209	43.8	65.7	45.7	36.4		29.1	46.4	54.6		5.2	7.8	9.0	
	Poor	1147	42.0	41.3	25.4	19.3		52.1	66.7	72.3		6.6	8.0	8.4	
Tumor size	<3 cm	1514	54.8	62.9	45.9	36.7	<0.001	30.5	45.3	53.1	<0.001	6.6	8.8	10.2	0.001
	≥3 cm	1248	45.2	49.7	32.1	24.2		45.4	62.1	68.3		4.9	6.7	7.4	
T stage (8th)	I	397	14.4	80.5	66.2	57.3	<0.001	13.3	24.5	30.4	<0.001	6.2	9.3	12.3	<0.001
	II	1177	42.6	68.8	51.5	42.1		24.9	39.8	4.7		6.2	8.7	10.2	
	III	1101	39.9	38.7	19.6	13.0		56.0	73.9	80.4		5.2	6.5	6.6	
	IV	87	3.1	28.6	12.9	5.7		65.0	79.3	86.5		6.4	7.8	7.8	
LN metastasis	Absent	1892	68.5	60.8	46.2	38.1	<0.001	32.6	45.1	51.5	<0.001	6.5	8.7	10.4	<0.001
	Present	870	31.5	48.8	25.1	16.9		46.9	69.0	77.1		4.3	6.0	6.0	
N stage (8th)	Absent	1914	69.3	60.7	45.9	37.7	<0.001	32.8	45.3	51.9	<0.001	6.6	8.8	10.4	<0.001
	1–3 LNs	778	28.2	48.6	25.4	17.3		47.3	68.8	76.8		4.1	5.9	5.9	
	≥4 LNs	70	2.5	52.5	21.4	15.6		43.2	74.2	80.1		4.4	4.4	4.4	
LNR	≤ 0.077	2040	73.9	60.6	45.9	37.8	<0.001	32.8	45.3	51.9	<0.001	6.6	8.7	10.4	<0.001
	>0.077	722	26.1	53.7	27.2	18.4		42.1	66.9	75.7		4.2	5.9	5.9	
TNM stage (8th)	I	351	12.7	84.4	71.6	61.9	<0.001	8.8	18.1	24.3	<0.001	6.8	10.3	13.8	<0.001
	II	765	27.7	74.0	60.8	51.0		18.3	29.2	36.7		7.7	10.0	12.4	
	III	1646	59.6	53.3	30.3	21.6		41.0	62.0	70.5		5.7	7.7	7.9	
Surgery type	Cholecystectomy	123	4.5	86.6	73.1	64.2	<0.001	6.7	17.7	20.6	<0.001	5.5	9.2	12.1	0.035
	Hepatectomy	2639	95.5	55.6	37.9	29.6		38.5	54.3	61.6		5.8	7.8	8.9	
Radiotherapy	No	2251	81.5	60.5	45.9	36.2	0.077	33.5	45.4	53.3	0.345	6.0	8.7	10.5	0.002
	Yes	511	18.5	76.9	50.2	36.2		20.1	46.6	59.4		3.0	3.8	4.3	
Chemotherapy	No	1836	66.5	58.4	45.4	36.3	0.172	35.0	45.0	51.9	0.368	6.6	9.5	11.8	<0.001
	Yes	926	33.5	73.9	48.9	35.6		23.1	46.9	59.8		3.0	4.2	4.6	

*LN, lymph node*.

**Figure 2:**
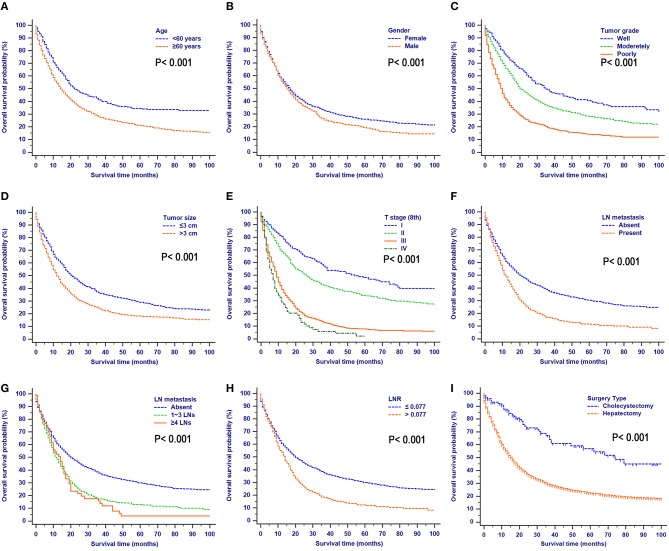
Overall survival rates stratified by patient characteristics: **(A)** age; **(B)** gender; **(C)** tumor grade; **(D)** tumor size; **(E)** T stage (8th); **(F)** LN metastasis; **(G)** N stage (8th); **(H)** LNR; **(I)** surgery type.

OS comparisons that were stratified by the aforementioned characteristics are shown in Figure 2. Patients with the characteristics of younger age, female gender, higher tumor grade, smaller tumor size, earlier T stages (8th), the absence of LN metastasis, earlier N stages (8th), lower LNR values, and earlier TNM stages (8th) all had significantly better OS.

### Univariate and Multivariate Analyses of Factors for OS and CSS

The median OS time for all the patients was 16 months (95% CI, 14.8–17.2 months), and the 1, 2, and 3-year OS rates for the whole cohort were 57.0, 39.3, and 31.1%, respectively. The 1, 2, and 3-year cumulative incidences of GBC death were 43.0, 60.7, and 68.9%, respectively. Three fourths of patients were randomly selected to form the training cohort (*n* = 2,072) and the remaining one fourth of patients (*n* = 690) was used to serve as an internal validation cohort. As shown in Table 2, the univariate analysis for OS revealed that age, gender, race, tumor grade, tumor size, T stage (8th), LN metastasis, N stage (8th), and LNR were significantly associated with survival. Additionally, the univariate competing risk analysis revealed that age, gender, race, tumor grade, tumor size, T stage (8th), LN metastasis, N stage (8th), and LNR were significantly associated with CSS. Chemotherapy and radiotherapy failed to act as risk factors for both OS and CSS.

**Table 2:** Univariate and multivariate analyses of survival in patients with gallbladder carcinoma.

**Characteristic**		**Overall survival**						**Cancer-specific survival**					
		**Univariate analysis**			**Multivariate analysis**			**Univariate analysis**			**Multivariate analysis**		
		**HR**	**95% CI**	***P***	**HR**	**95% CI**	***P***	**HR**	**95% CI**	***P***	**HR**	**95% CI**	***P***
Age (years)	<60/≥60	1.527	1.355–1.720	<0.001	1.701	1.498–1.931	<0.001	1.386	1.223–1.571	<0.001	1.590	1.389–1.819	<0.001
Gender	Male/female	1.132	1.021–1.255	0.018	1.178	1.059–1.311	0.003	1.138	1.018–1.272	0.023	1.209	1.070–1.358	0.001
Race	White/Black/Others	0.913	0.851–0.979	0.011	0.942	0.875–1.013	0.106	0.924	0.857–0.996	0.039	0.950	0.878–1.028	0.200
Tumor grade	Well/Moderate/Poor	1.634	1.522–1.755	<0.001	1.358	1.257–1.466	<0.001	1.756	1.623–1.899	<0.001	1.411	1.296–1.535	<0.001
Tumor size	<5 cm/≥5 cm	1.417	1.290–1.556	<0.001	1.126	1.018–1.245	0.021	1.550	1.401–1.716	<0.001	1.171	1.050–1.306	0.005
T stage (8th)	T1/T2/T3/T4	1.932	1.809–2.064	<0.001	1.669	1.543–1.804	<0.001	2.183	2.032–2.346	<0.001	1.848	1.695–2.015	<0.001
LN metastasis	Absent/Present	1.624	1.474–1.790	<0.001	0.824	0.559–1.217	0.331	1.818	1.639–2.017	<0.001	0.772	0.510–1.167	0.220
N stage (8th)	Absent/1–3 LNs/≥4 LNs	1.477	1.359–1.605	<0.001	0.967	0.739–1.264	0.806	1.619	1.484–1.767	<0.001	0.985	0.747–1.299	0.916
LNR (binary variable)	≤ 0.077/>0.077	1.508	1.358–1.674	<0.001	0.913	0.579–1.440	0.697	1.675	1.499–1.872	<0.001	0.982	0.604–1.597	0.942
LNR (continuous variable)		1.796	1.588–2.031	<0.001	1.737	1.308–2.305	<0.001	2.013	1.768–2.292	<0.001	1.782	1.322–2.402	<0.001
Chemotherapy	No/Yes	0.867	0.528–1.768	0.088			NI	0.875	0.652–1.759	0.074			NI
Radiotherapy	No/Yes	0.752	0.651–1.325	0.158			NI	0.835	0.772–1.872	0.192			NI

*LN, lymph node; LNR, lymph node ratio; HR, hazard ratio; CI, confidence interval; NI, not included*.

The OS and CSS multivariate analyses were constructed based on the variables identified through the univariate analyses (Table 2). The independent prognostic factors of OS identified via the multivariate analysis included age, gender, tumor grade, tumor size, T stage (8th), and LNR (continuous variable). Moreover, the proportional sub-distribution hazard assumption was held for the variables used for the CSS analysis. Increasing age was related to a decreased probability of GBC-specific survival. Gender was also predictive of CSS, with a significant sub-distribution hazard ratio (HR) of 1.209. In addition, tumor grade, tumor size, T stage (8th), and LNR (continuous variable) were all significant independent predictors of CSS.

### Construction and Validation of Nomograms for OS and CSS

**Figure 3:**
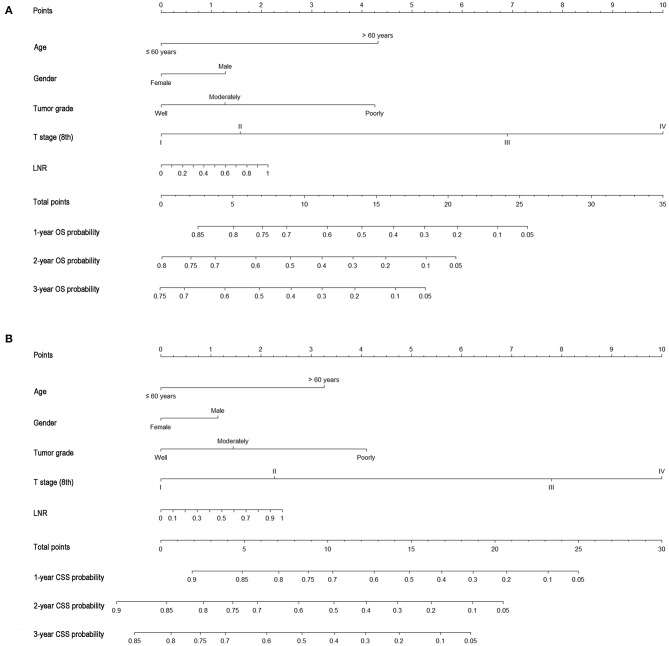
Nomograms predicting 1-, 2-, and 3-year OS **(A)** and CSS **(B)** of patients with gallbladder carcinoma.

**Figure 4:**
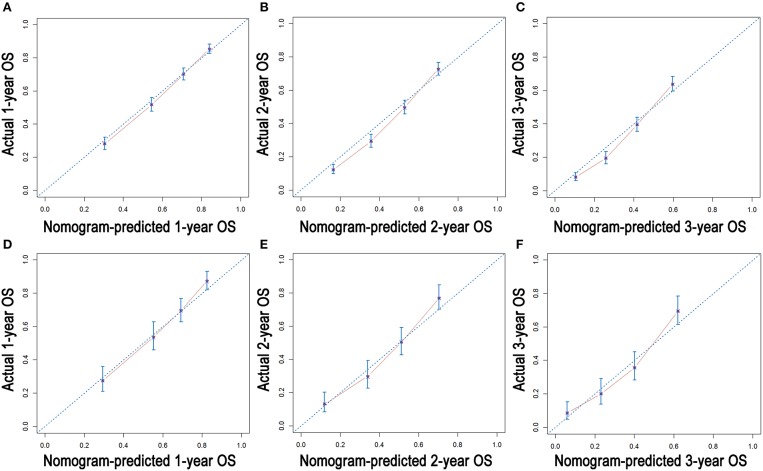
Calibration plots of the nomogram for 1-, 2-, and 3-year OS prediction of the training cohort **(A–C)** and internal validation cohort **(D–F)**.

**Figure 5:**
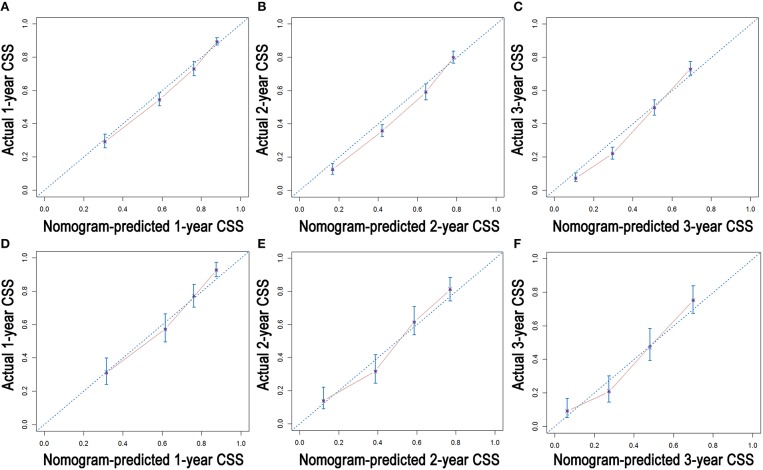
Calibration plots of the nomogram for 1-, 2-, and 3-year CSS prediction of the training cohort **(A–C)** and internal validation cohort **(D–F)**.

As shown in Figure 3, the nomograms for predicting OS were established with a C-index of 0.704 (95% CI, 0.690–0.718) for the training cohort, demonstrating good accuracy for OS prediction. The C-indexes of the nomogram based on internal and external cohorts were also higher than those based on the 8th TNM stage (Table 3). Calibration was illustrated by plots showing high agreement between the predicted and actual survival in both the training and internal validation cohorts (Figure 4). The external validation with the SYSUCC cohort of the established nomograms also demonstrated fairly optimal agreements between the actual and predicted survival probabilities (Figure S1). Additionally, in terms of CSS prediction, relatively good nomogram accuracy for CSS prediction was observed with a C-index of 0.732 (95% CI, 0.718–0.755) for the training cohort. The calibration plots also confirmed optimal agreement between CSS prediction and the actual observations for training, internal validation cohort (Figure 5), and external validation cohort (Figure S1). Moreover, compared with the 8th edition TNM staging system, the nomograms had significantly higher values of C-indexes, showing more powerful efficiency of discrimination for CSS prediction (Table 3).

**Table 3:** C-indexes for the nomograms and TNM stage systems in patients with gallbladder carcinoma.

**Survival**		**Training cohort**	***P***	**Internal validation cohort**	***P***	**Internal validation cohort**	***P***
Overall survival	Nomogram	0.704 (95% CI, 0.690–0.718)	<0.001	0.718 (95% CI, 0.692–0.744)	0.002	0.696 (95%CI, 0.663–0.729)	0.03
	8th TNM stage	0.660 (95% CI, 0.646–0.673)		0.674 (95% CI, 0.647–0.701)		0.664 (95%CI, 0.628–0.700)	
Cancer-specific survival	Nomogram	0.732 (95% CI, 0.718–0.755)	<0.001	0.745 (95% CI, 0.718–0.771)	0.013	0.722 (95%CI, 0.689–0.755)	0.045
	8th TNM stage	0.694 (95% CI, 0.680–0.708)		0.710 (95% CI, 0.683–0.737)		0.692 (95%CI, 0.656–0.728)	

### Comparison of the Values of Area Under the ROC Curve

**Figure 6:**
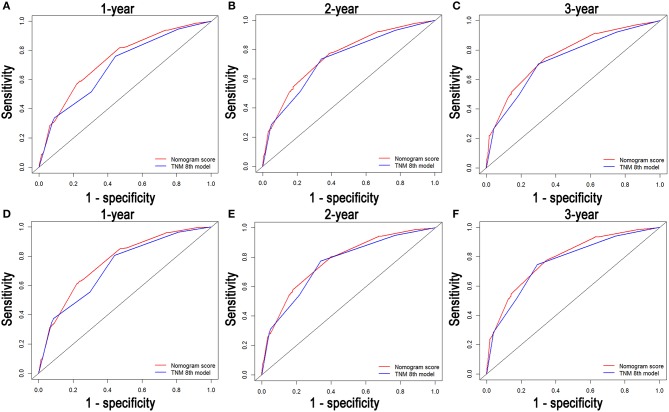
Comparison of the ROC curves of the nomogram and the TNM stage systems for 1-, 2-, and 3-year OS prediction **(A–C)** and CSS prediction **(D–F)**.

Comparisons of the discriminatory capacity between the nomograms and the 8th TNM staging system are shown in Figure 6. For the training cohort, the area under ROC curve (AUC) values of the nomogram for predicting the 1-, 2-, and 3-year OS rates were 0.732, 0.752, and 0.765, and 0.704, 0.731, and 0.736 for the 8th TNM staging system, respectively. With regard to the prediction of the 1-, 2-, and 3-year CSS rates, higher AUC values were observed for the nomogram compared with the 8th TNM staging system. Similar results were also obtained for the internal and external validation cohort (Table 4).

**Table 4:** Comparison of the AUC values between nomograms and TNM stages.

**Patients**		**Overall survival**			**Cancer-specific survival**		
		**1 year**	**2 years**	**3 years**	**1 year**	**2 years**	**3 years**
Whole cohort	Nomogram	0.737	0.756	0.766	0.761	0.773	0.787
	8th TNM stage	0.707	0.737	0.738	0.738	0.759	0.765
Training cohort	Nomogram	0.732	0.752	0.765	0.753	0.768	0.783
	8th TNM stage	0.704	0.731	0.736	0.733	0.753	0.762
Internal validation cohort	Nomogram	0.752	0.768	0.769	0.784	0.789	0.795
	8th TNM stage	0.715	0.753	0.744	0.754	0.777	0.772
External validation cohort	Nomogram	0.712	0.734	0.733	0.743	0.751	0.761
	8th TNM stage	0.680	0.729	0.711	0.716	0.745	0.737

## Discussion

In this study, we evaluated the mortality and survival rates of patients with GBC after surgery. As the most aggressive cancer of the biliary tract (26), GBC alone was evaluated in previous studies. Moreover, such studies were mainly single-center studies with small sample sizes. The accuracy of the conclusions regarding the clinical characteristics and survival outcomes of GBC were therefore not convincing. In this study, well-calibrated prognostic nomograms were constructed to predict OS and CSS in patients with GBC after surgery. Moreover, compared with the 8th TNM staging system, the superior discriminative power of the nomograms was confirmed by the higher AUC values for patients with GBC after surgery.

It was shown that age may have a significant impact on survival (27). A similar negative effect of increasing age on the survival of patients with GBC after surgery was observed in this study. Moreover, this negative effect was more obvious for OS than CSS. Similar to the results of previous studies, the present study also suggested that non-cancer-specific death, likely from age-related comorbid conditions, was an important competing risk event in older patients (17, 28). Thus, it is important to evaluate surgery tolerance among older patients and to consider age when prognoses are developed for patients with GBC after surgery.

In the nomograms for predicting OS and CSS, the other predictors of decreasing probabilities of survival for patients with GBC after surgery included gender, advanced tumor grades, more advanced T stages, and higher LNR levels. Because cholecystectomy and hepatectomy combined with lymphadenectomy were mainly performed for patients with T1a and T1b-T4 diseases, respectively, to avoid multicollinearity, surgery type was not included into the univariate and multivariate analyses (29). Similar results have also been reported in other cancers (28, 30) where male patients and patients with more advanced TNM stages were associated with a higher risk of cancer-specific mortality. Furthermore, tumor grade, which is also an inherent characteristic of tumors and has been shown in many previous studies (31, 32) to be an independent prognostic factor in patients with GBC, was considered when the prognosis was evaluated in patients with GBC after surgery in the present study. The inclusion of the additional variables in the nomograms led to increased accuracy in survival prediction using nomograms compared with the 8th edition TNM staging system. Additionally, their inclusion may partly explain the superior power of the nomograms in predicting OS and CSS. However, chemotherapy and radiotherapy failed to show significant associations with survival in this study. Similar to previous studies, limited success was achieved from adjuvant therapies for GBC (6). Moreover, the absence of unified regimens of chemotherapy and radiotherapy would partly contribute to the role as a non-significant risk factor of adjuvant therapy in patients with GBC after surgery. With the development of chemotherapy and radiotherapy for GBC, the importance of adjuvant therapies in the treatment of resectable GBC would truly increase gradually.

Patient counseling and decision-making are based on the prognoses estimated from individual risk profiles. Recently, competing risk nomograms have been developed for many tumors, such as lung cancer (28), melanoma (33), breast cancer (34), and prostate cancer (35). However, there was no specific competing risk staging for GBC. The present study was therefore the first to evaluate prognostic factors based on a competing risk analysis model for patients with GBC after surgery. The present nomograms showed good discrimination with higher C-indexes and AUC values in both the training and validation cohorts compared with 8th edition TNM staging system. Well-corresponded calibration plots were also observed for the prediction of OS and CSS using the nomograms. The inclusion of additional variables might contribute to the elevation of predictive power of the established nomograms, compared to the TNM stage system. More importantly, a more generalizable conclusion could be drawn based on the large cohort in this study. The nomograms, which comprise a few easily obtained predictors, could help doctors make accurate individual prognosis estimates and select groups of patients with different risks of decreased survival after surgery. Patients with high risks of decreased survival could benefit more from adjuvant therapies, including chemotherapy and radiotherapy. Therefore, with this easily used predictive system, diverse risk factors of patients could be assessed by doctors more objectively and precisely. A more homogeneous prognosis estimation would contribute to more specialized personal treatment finally.

This study had several limitations. First, some potential prognostic factors, such as carbohydrate antigen 19-9 (CA19-9), carcinoembryonic antigen (CEA), and vascular invasion, were not available in the SEER dataset and were therefore not included in the nomograms as the evaluation of such variables could not be carried out in this study. Second, although a large cohort and single-center external validation were available for this study, further external validations based on more large-scale cohorts are still needed to estimate the accuracy of the model.

In conclusion, we developed a novel staging system based on a large population-based cohort to estimate the cumulative incidences of OS and CSS for patients with GBC after surgery. The well-calibrated nomograms may facilitate highly tailored patient management in clinical practice.

## Data Availability Statement

The datasets generated for this study are available on request to the corresponding author.

## Author Contributions

XL was responsible for conception, design, quality control of this study, reviewed, and edited the manuscript. CH, ZC, and YZ performed the study selection, data extraction, statistical analyses, and were major contributors in writing the manuscript and contributed in classification criteria discussion. CH and ZC participated in studies selection and statistical analyses. All authors have read and approved the final version of the manuscript.

### Conflict of Interest

The authors declare that the research was conducted in the absence of any commercial or financial relationships that could be construed as a potential conflict of interest.
