# Supplementary material for: Prognostic Model to Predict Cancer-Specific Survival for Patients With Gallbladder Carcinoma After Surgery: A Population-Based Analysis
Source: Front Oncol. 2019 Dec 12;9:1329. doi: 10.3389/fonc.2019.01329 (PMC6920125; doi:10.3389/fonc.2019.01329)
Supplement: Supplementary file 1 [file Data_Sheet_1.pdf]

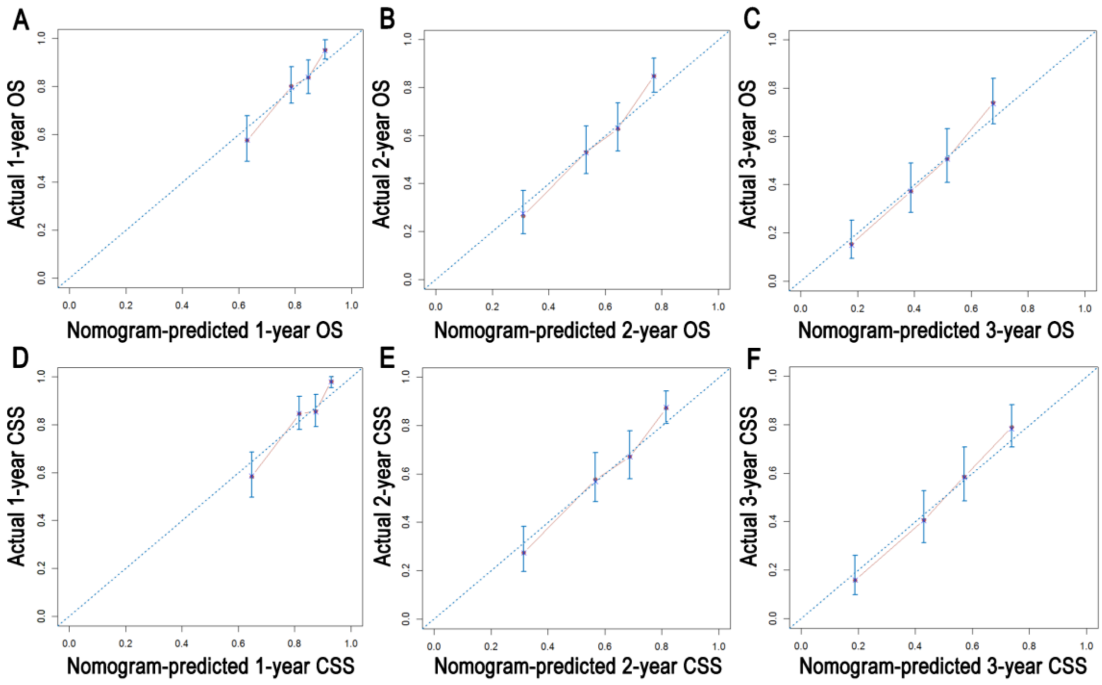

Figure S1 | Calibration plots of the nomogram for 1-, 2- and 3-year OS (A, B, C) and CSS (D, E, F) prediction of the external validation cohort.
